# Simulation-Based Medical Education and Training Enhance Anesthesia Residents' Proficiency in Erector Spinae Plane Block

**DOI:** 10.3389/fmed.2022.870372

**Published:** 2022-04-08

**Authors:** Vito Torrano, Francesco Zadek, Dario Bugada, Gianluca Cappelleri, Gianluca Russo, Giulia Tinti, Antonio Giorgi, Thomas Langer, Roberto Fumagalli

**Affiliations:** ^1^Department of Medicine and Surgery, University of Milan-Bicocca, Monza, Italy; ^2^Department of Anesthesia and Intensive Care Medicine, Niguarda Ca' Granda, Milan, Italy; ^3^Department of Emergency and Critical Care Medicine, Azienda Socio Sanitaria Territoriale Papa Giovanni XXIII, Bergamo, Italy; ^4^Anesthesia and Intensive Care Unit, Policlinico di Monza, Monza, Italy; ^5^Department of Emergency and Urgency, Azienda Socio Sanitaria Territoriale Lodi, Lodi, Italy

**Keywords:** ultrasound-guided regional anesthesia, medical education, high fidelity simulation, resident training, improved proficiency, erector spinae plane (ESP) block

## Abstract

**Background:**

Advances in regional anesthesia and pain management led to the advent of ultrasound-guided fascial plane blocks, which represent a new and promising route for the administration of local anesthetics. Both practical and theoretical knowledge of locoregional anesthesia are therefore becoming fundamental, requiring specific training programs for residents. Simulation-based medical education and training (SBET) has been recently applied to ultrasound-guided regional anesthesia (UGRA) with remarkable results. With this in mind, the anesthesia and intensive care residency program of the University of Milano-Bicocca organized a 4-h regional anesthesia training workshop with the BlockSim^®^ (Accurate Srl, Cesena) simulator. Our study aimed to measure the residents' improvement in terms of reduction in time required to achieve an erector spinae plane (ESP) block.

**Methods:**

Fifty-two first-year anesthesia residents were exposed to a 4-h training workshop focused on peripheral blocks. The course included an introductory theoretical session held by a locoregional anesthetist expert, a practical training on human models and mannequins using Onvision^®^ (B. Braun, Milano) technologies, and two test performances on the BlockSim simulator. Residents were asked to perform two ESP blocks on the BlockSim: the first without previous practice on the simulator, the second at the end of the course. Trainees were also also asked to complete a self-assessment questionnaire.

**Results:**

The time needed to achieve the block during the second attempt was significantly shorter (131 [83, 198] vs. 68 [27, 91] s, *p* < 0.001). We also observed a reduction in the number of needle insertions from 3 [2, 7] to 2 [1, 4] (*p* = 0.002), and an improvement aiming correctly at the ESP from 30 (58%) to 46 (88%) (*p* < 0.001). Forty-nine (94%) of the residents reported to have improved their regional anesthesia knowledge, 38 (73%) perceived an improvement in their technical skills and 46 (88%) of the trainees declared to be “satisfied/very satisfied” with the course.

**Conclusions:**

A 4-h hands-on course based on SBET may enhance first-year residents' UGRA ability, decrease the number of punctures and time needed to perform the ESP block, and improve the correct aim of the fascia.

## Introduction

Over the past few years, technological innovations in anatomical-based ultrasound imaging allowed significant advancements in regional anesthesia. Ultrasound-guided regional anesthesia (UGRA) represents the gold standard for peripheral regional blocks, including fascial blocks (1–3).

Ultrasound guidance is directly linked to the widespread interest for fascial plane blocks, according to the motto: “if we see it, we can block it” (4–6). The diffusion of fascial plane blocks relies on their simplicity and/or safety when compared to other approaches, such as neuraxial/peripheral nerve blocks. Fascial plane blocks represent a new route for delivering local anesthetics to various anatomic sites and play a growing role in multimodal analgesia and opioid-sparing pathways (2, 5).

Due to the abovementioned features, both residents and specialists of anesthesia and other disciplines are increasingly applying these blocks (7, 8). To correctly perform a UGRA procedure, skills such as hand-eye coordination, image acquisition and anatomical interpretation must be learned (9, 10). Wide variability in UGRA skills has been described among residents (9). Therefore, teaching and training programs in regional anesthesia are fundamental for improving the UGRA skills of future (and current) anesthetists. However, a standardized curriculum has not yet been proposed (9).

Simulation-based medical education and training (SBET) are increasingly applied in several fields. In the last few years, simulation has also been applied to UGRA, and several advantages have been observed: the creation of a low-stress learning condition without time pressure; individualized expert feedback; learning curve shortening; opportunity of repetitive practice in a safe environment. All these aspects likely improve block success (9–11), as suggested by recent qualitative systematic reviews (10).

Currently, few Anesthesia and Intensive Care societies are equipped with simulators. Among these, both the Italian Society of Anesthesiology, Analgesia, Resuscitation, and Intensive Care (SIAARTI) and the American Board of Anesthesiology have started integrating SBET in their training programs to enhance trainees' technical and non-technical skills (12–14).

In this framework, the Anesthesia and Intensive Care Residency Program of the University of Milan-Bicocca, organized a 1-day simulation training in UGRA. The present study aimed to characterize quantitatively and qualitatively the improvement in UGRA of first-year anesthesia residents after a 1-day simulation training.

## Materials and Methods

### Study Design

The first-year anesthesiology residents of Milan-Bicocca University were enrolled. In order to accommodate all trainees, the participants were divided into three aliquots of 15-20 residents and the study took place over the course of 3 separate training sessions that lasted 4 h each.

**Figure 1:**
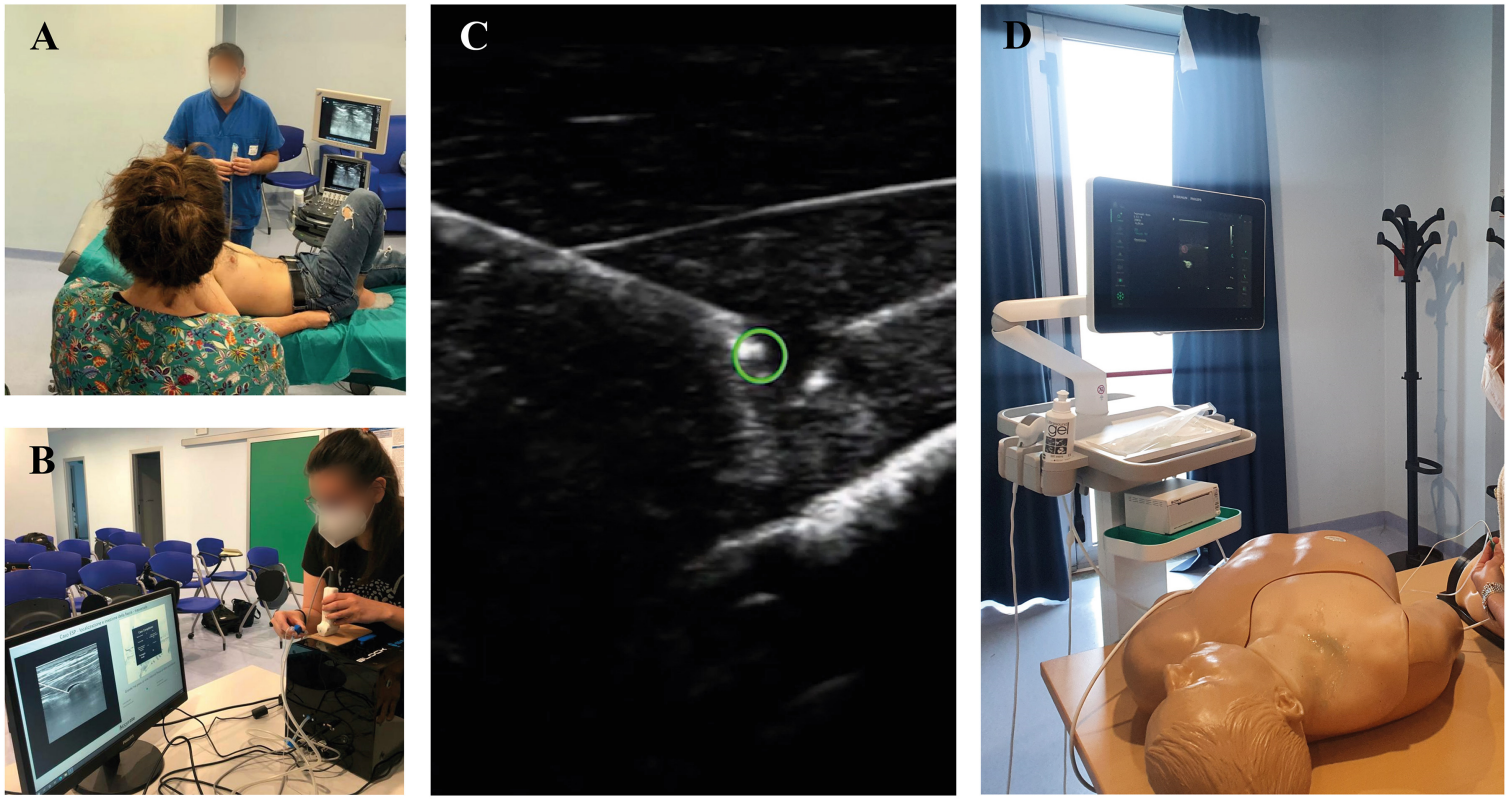
**(A)** Hands-on session; **(B)** the BlockSim^®^ simulator; **(C)** Onvision^®^ Needle Tip Tracking; **(D)** Regional anesthesia mannequin simulation station.

The course, structured in four parts, focused on fascial plane blocks and, in particular, on erector spinae plane (ESP) block. The first part included a 1-h introductory theoretical training performed by an experienced anesthesiologist on a member of school staff to explain probe management and sonoanatomy (Figure 1A). Afterward, one at a time, each resident was randomly called to perform an ESP block with the BlockSim (Accurate Srl, Cesena) without any previous practice (Figure 1B). The simulator was isolated so that nobody could see their peers executing the block. The third part consisted of 2 h of training on three different tasks: probe-handling on human models; needle tip detection and tracking using Onvision (B. Braun S.p.A, Milano) on mannequins (Figures 1C,D, respectively); performance of fascial blocks other than ESP on BlockSim. Lastly, at the end of the 4-h course, each resident repeated the ESP block on BlockSim.

### Employed Teaching Technology

During the training, the Onvision system was employed to exercise needle tip detection and probe handling. This innovative technology, combined with the ultrasound machine, is able to project the position of the tip on the ultrasound image, displayed as a colored circle. Moreover, the color of the circle is changed from red (out of plane) to green (optimal alignment), giving immediate feedback to the operator of the correct alignment of the needle tip with the probe (15, 16).

In addition, the BlockSim simulator, a high-fidelity system for ultrasound-guided fascial blocks based on real ultrasound images, was used. This simulator allows performing multiple fascial blocks, including abdominal wall blocks (quadratus lumborum block, transversus block), anterior thoracic wall blocks [Pectoralis nerve (PECS) 1, PECS 2, serratus plane block, and parasternal block], and posterior thoracic wall blocks (ESP, paravertebral block). Moreover, at the end of the scenario, the simulator reports information regarding the performance of the block (see below) (17).

### Data Collection

Each participant was provided with a unique identifying number. The following data were retrieved from the BlockSim for both ESP attempts: the correct aim at the fascia (yes/no), the time needed for procedure achievement (expressed in seconds), and the number of needle insertions (17). Additionally, a questionnaire was emailed to each participating resident to retrieve demographic data, previous experience regarding fascial blocks, course evaluation, and self-assessment about conceptual and practical locoregional skills, both before and after the workshop. The English translation of the questionnaire is reported in Supplementary Table 1.

### Statistical Analysis

Continuous data are expressed as mean ± standard deviation or as a median and interquartile range, while categorical data are expressed as count and percentage. The difference in time needed to perform the ESP and the number of needle insertions prior to and after the course was assessed using the Signed Rank Test. Differences between categorical variables were assessed using the Chi-Square or Fisher's exact test, as appropriate.

The primary endpoint was the reduction in time (expressed in seconds) required to achieve the ESP block assessed with the BlockSim after the one-day training. Secondary endpoints were the reduction of needle insertions, aim at the fascia, self-assessment of theoretical and practical skills regarding UGRA after the course. In addition, the study population was divided in two cohorts, according to the declared prior experience in ESP performance (Group 1 = residents have performed at least 1 ESP block; Group 2 = residents have never performed an ESP block), in order to evaluate the impact of prior experience on block performance and improvement. No a priori sample size calculation was performed. All first-year residents attending the training were enrolled. Statistical significance was defined as *p* <0.0 5. Analysis was performed with SigmaPlot v.12.0 (Systat Software, San Jose, CA).

## Results

Fifty-two first-year residents in Anesthesia and Intensive Care Medicine participated to the study. The median age of the residents was 27 ± 1 years. The average time of training in the operating room at the time of the course was 6 months. On the day of the course, 49 (94%) residents declared to have previously observed the performance of a fascial block, while 43 (83%) residents had already performed at least one fascial block during the clinical training. Twelve (23%) residents had performed at least one ESP in clinical practice (Group 1), while the remaining 40 (77%) residents never performed the ESP block (Group 2). The results of self-evaluation from the questionnaire are reported in Table 1.

**Table 1:** Residents questionnaire self-evaluation.

	**Null level**	**Low level**	**Good level**	**Excellent level**
Practical knowledge of peripheral regional anesthesia before the course—*n* (%)	8 (15)	23 (44)	20 (38)	1 (2)
Theoretical knowledge of ESP block before the course—*n* (%)	11 (21)	34 (65)	7 (13)	0 (0)

*This table represents the declared self-evaluation of the resident on their experience with regional anesthesia and ESP block before the course. ESP, erector spinae plane*.

After the 4-h training, 40 (77%) residents reduced the time needed to perform the ESP block. The time needed to perform the block decreased 74 [2, 136] seconds in the whole study population. Thirty-four (65%) residents decreased the number of needle insertions needed to achieve the block, with an overall median reduction of 2 [0, 4] punctures. Lastly, their technical skills to correctly aim at the ESP improved from 30 (58%) to 46 (88%) (*p* < 0.001) (Table 2). When dividing the population according to their prior experience in performing the ESP block, we did not observe significant differences regarding the tasks tested both at the first and second ESP attempt at the BlockSim (Table 3).

**Table 2:** Performance of the first and second attempts at the BlockSim in the overall resident population.

	**First attempt (*n* = 52)**	**Second attempt (*n* = 52)**	***P***
Correctly aimed at the target with the needle—*n* (%)	30 (58)	46 (88)	<0.001
Number of punctures—*n*	3 [2, 7]	2 [1, 4]	0.002
Time to target—s	131 [83, 198]	68 [27, 91]	<0.001

*This table reports the performance before and after the 4-h course at the BlockSim in the overall resident population*.

**Table 3:** Performance at the first and second attempt at the BlockSim dividing the population for previous experience in performing ESP Block.

	**Group 1** **(*n* = 12)**	**Group 2** **(*n*= 40)**	***P***
**First attempt**			
Correctly aimed at the target with the needle—*n* (%)	6 (50)	24 (60)	0.54
Number of punctures	6 [3, 8]	3 [2, 6]	0.22
Time to target—s	150 [126, 227]	120 [77, 188]	0.08
**Second attempt**			
Correctly aimed at the target with the needle—*n* (%)	11 (92)	35 (88)	0.58
Number of punctures	2 [1, 3]	3 [1, 4]	0.35
Time to target—s	50 [17, 92]	72 [29, 91]	0.28

*This table reports the performance before and after the 4-h course at the BlockSim, dividing the population based on the declared experience. Group 1, residents who have performed at least 1 ESP block before the course; Group 2, the residents who have never performed an ESP block before the 4-h course*.

After a 1-day UGRA training workshop, 49 (94%) of the residents declared to have improved their knowledge regarding peripheral regional anesthesia, and 38 (73%) felt improved their practical skills. Forty-six (88%) of the participants declared to be “satisfied” or “very satisfied” with the workshop.

## Discussion

This study suggests that SBET might be very useful to improve anesthesia residents' theoretical and practical UGRA skills. The BlockSim recorded a statistically significant improvement both in the execution time and the block's quality, intended as the number of needle attempts and correct target injection.

The number of locoregional anesthesia procedures is steadily increasing (1, 3). Moreover, the widespread use of ultrasound guidance favored the introduction of new fascial plane blocks, ultimately increasing the sonoanatomic knowledge required to perform the procedures. Given the increasing number of residents and the abovementioned introduction of new blocks, it is a continuous challenge for anesthesia residency programs to guarantee an adequate UGRA competency to their trainees. The use of SBET is a possible intervention to improve the exposure to UGRA and develop UGRA skills. Indeed, during SBET, residents can perform multiple blocks in low-stress conditions without the risk of harm to the patient and might receive individualized feedback from experts in the field. Moreover, simulators allow the residents to exercise and perform peripheral blocks less frequently encountered during the clinical training (18).

Several studies have demonstrated the efficacy of SBET in reducing the learning curve of nerve blocks and improving their success rate (10, 11, 14, 19). Niazi et al. showed that a short 1-h practice session on a low-fidelity simulation model, in addition to conventional teaching, improved the overall success rate of UGRA in novice residents (19).

A second useful feature of SBET is the ability to objectively measure the performance and monitor the improvements for each simulated block. Consequently, the simulator allows to individually analyze the different technical skills required for a successful and safe procedure.

## Conclusions

In line with the available literature, our study reveals that a 4-h training based on SBET may enhance proficiency in UGRA in first-year anesthesia residents reducing the number of punctures, improving the correct aim of the fascia, and decreasing the time needed to perform the ESP block.

Pearson's law states: “When performance is measured, performance improves. When performance is measured and reported, the rate of improvement accelerates.”

## Data Availability Statement

The original contributions presented in the study are included in the article/Supplementary Material, further inquiries can be directed to the corresponding author/s.

## Ethics Statement

Ethical review and approval was not required for the study on human participants in accordance with the local legislation and institutional requirements. The patients/participants provided their written informed consent to participate in this study. Written informed consent was obtained from the individual(s) for the publication of any potentially identifiable images or data included in this article.

## Author Contributions

VT, TL, GC, DB, GR, and RF contributed to conception and design of the study. AG organized the database. FZ and TL performed the statistical analysis. GT wrote the first draft of the manuscript. All authors contributed to manuscript revision, read, and approved the submitted version.

## Conflict of Interest

The authors declare that the research was conducted in the absence of any commercial or financial relationships that could be construed as a potential conflict of interest.

## Publisher's Note

All claims expressed in this article are solely those of the authors and do not necessarily represent those of their affiliated organizations, or those of the publisher, the editors and the reviewers. Any product that may be evaluated in this article, or claim that may be made by its manufacturer, is not guaranteed or endorsed by the publisher.
