# Supplementary material for: Simulation-Based Medical Education and Training Enhance Anesthesia Residents' Proficiency in Erector Spinae Plane Block
Source: Front Med (Lausanne). 2022 Apr 8;9:870372. doi: 10.3389/fmed.2022.870372 (PMC9024057; doi:10.3389/fmed.2022.870372)
Supplement: Supplementary file 1 [file Table_1.docx]

**Simulation-based medical education and training enhance anesthesia residents’ proficiency in erector spinae plane block.**

Vito Torrano^1,2^, Francesco Zadek^1^, Dario Bugada^3^, Gianluca Cappelleri^4^, Gianluca Russo^5^,
Giulia Tinti^1^, Antonio Giorgi^1^, Thomas Langer^1,2^, Roberto Fumagalli^1,2^

^1^ Department of Medicine and Surgery, University of Milan-Bicocca, Monza, Italy

^2^ Department of Anesthesia and Intensive Care Medicine, Niguarda Ca’ Granda, Milan, Italy

^3^ Department of Emergency and Critical Care Medicine, Azienda Socio Sanitaria Territoriale Papa Giovanni XXIII, Bergamo, Italy

^4^ Anesthesia and Intensive Care Unit, Policlinico di Monza, Monza, Italy

^5^ Department of Emergency and Urgency, Azienda Socio Sanitaria Territoriale Lodi, Lodi, Italy

**Corresponding Author:**

Thomas Langer, MD; Department of Medicine and Surgery, University of Milan-Bicocca, Monza, Italy; Department of Anesthesia and Intensive Care Medicine, Niguarda Ca’ Granda, Milan, Italy, Italy. tel. +39 02 64448580; fax: +39 02 2.6444455; email: Thomas.Langer@unimib.it

**Supplementary Table 1: The English translation of the questionnaire**

| **Questionnaire** |
| --- |
|  |
| Name |
|  |
| Surname |
|  |
| Age |
|  |
| How satisfied are you with the locoregional course on a simulator? |
| - Very unsatisfied |
| - Unsatisfied |
| - Satisfied |
| - Very satisfied |
|  |
| How would you describe your knowledge of locoregional anesthesia before the course? |
| - Null |
| - Scarce |
| - Good |
| - Excellent |
|  |
| Do you think that the locoregional anesthesia course on a simulator has enriched your knowledge in this area? |
| - Absolutely not |
| - A little |
| - A lot |
| - Enormously |
|  |
| Have you ever seen a fascial plane block performed (on a patient)? |
| - No |
| - Yes |
|  |
| Have you ever performed a fascial plane block yourself (on a patient)? |
| - No |
| - Yes |
|  |
| Have you ever seen an ESP block performed (on a patient)? |
| - No |
| - Yes |
|  |
| Have you ever performed an ESP block (on a patient)? |
| - No |
| - Yes |
|  |

| How would you define your theoretical knowledge of ESP block before the course? |
| --- |
| - Null |
| - Scarce |
| - Good |
| - Excellent |
|  |
| How would you define your theoretical knowledge of ESP block after the course? |
| - Null |
| - Scarce |
| - Good |
| - Excellent |
|  |
| How would you define your practical skills of locoregional anesthesia before the course? |
| - Null |
| - Scarce |
| - Good |
| - Excellent |
|  |
| How would you define your practical skills of locoregional anesthesia after the course? |
| - Null |
| - Scarce |
| - Good |
| - Excellent |
